# The distribution of incidence rates of cardiovascular diseases in the elderly and the relationship between dietary patterns and cardiovascular risk

**DOI:** 10.7717/peerj.20768

**Published:** 2026-02-27

**Authors:** Qi Yang, Juan Liu

**Affiliations:** 1 Department of Medical Services, Huangshi Central Hospital, Affiliated Hospital of Hubei Polytechnic University, Huangshi, Hubei, China; 2 Department of Science and Education, Huangshi Central Hospital, Affiliated Hospital of Hubei Polytechnic University, Huangshi, Hubei, China

**Keywords:** Cardiovascular disease, Elderly, Dietary patterns, Ischemic heart disease, Metabolic markers, Risk factors

## Abstract

**Background:**

Cardiovascular diseases (CVD) were leading causes of morbidity and mortality in the elderly. This study investigates the distribution of CVD incidence among older adults and examines the relationship between dietary patterns and associated risks.

**Methods:**

A retrospective analysis was conducted involving 2,568 patients aged 65 and older hospitalized between 2022 and 2024. Out of these, 298 patients were selected based on specific inclusion criteria for cardiovascular risk assessment using the China-PAR model. Participants were stratified into low-risk and high-risk groups for CVD based on a 5% threshold. Detailed dietary intake was assessed using the European Prospective Investigation into Cancer and Nutrition (EPIC) questionnaire, and blood samples were analyzed for biochemical markers. Statistical analyses including logistic regression were applied to examine dietary influences on CVD risk.

**Results:**

Ischemic heart disease was most prevalent (37.83%) among the elderly, with increased incidence in males. Higher intakes of fruits, vegetables, legumes, cereals, and fish correlated with reduced CVD risk. Conversely, consumptions of meat, edible oil, and alcohol were associated with heightened risk. The high-risk group exhibited poorer markers of cardiometabolic health and increased intake of energy, saturated fatty acids (SFA), and cholesterol. Metabolite analyses revealed elevated trimethylamine N-oxide (TMAO) and secondary bile acids in high-risk individuals.

**Conclusion:**

Higher age and male gender were associated with increased CVD risk, exacerbated by specific dietary patterns. Diets rich in plant-based foods and low in animal products and alcohol effectively reduced CVD risk factors. These findings underscore the potential of dietary interventions to improve cardiovascular health in the elderly.

## Introduction

Cardiovascular diseases (CVD) remain the leading cause of morbidity and mortality worldwide, and their impact was particularly pronounced in the elderly population (Bułdak, 2022; Lavie, 2022; Mensah, Roth & Fuster, 2019). Aging was associated with numerous physiological changes that elevate the risk for cardiovascular conditions, including increased arterial stiffness, endothelial dysfunction, and altered autonomic regulation (Ciumărnean et al., 2021; Evans, Sano & Walsh, 2020; Noale, Limongi & Maggi, 2020). Despite advancements in medical treatment and preventive measures, the incidence of CVD in older adults continues to rise, underscoring the need for effective strategies to curb this trend. One such strategy involves the identification of modifiable risk factors, particularly those related to lifestyle choices, such as diet.

Epidemiological studies have repeatedly demonstrated the crucial role of diet in the development and progression of CVD (Diab et al., 2023; Dyńka et al., 2023; Fukumoto, 2021). Nutritional status and dietary patterns have been implicated in influencing cardiovascular health through multiple mechanisms, including lipid metabolism regulation, oxidative stress reduction, and inflammation modulation (Melhem & Taleb, 2021; Petersen & Kris-Etherton, 2021; Salehin et al., 2023). The recognition of dietary patterns that can mitigate cardiovascular risk was especially pertinent for older adults, who might benefit significantly from dietary adjustments given their vulnerability to age-related health deterioration.

In recent decades, research has increasingly focused on the relationship between specific dietary patterns and health outcomes, moving away from examining individual nutrients to considering overall dietary intake (Sharifi-Rad et al., 2020). Diets rich in fruits, vegetables, legumes, cereals, and fish have consistently been associated with reduced cardiovascular risk, largely due to their high content of nutrients with antioxidative and anti-inflammatory properties (Chareonrungrueangchai et al., 2020). Conversely, diets characterized by high consumption of saturated fats, trans fats, and cholesterol—often found in processed meats and refined oils—have been associated with increased cardiovascular risk (Jeong et al., 2023).

This study investigates the distribution of CVD incidence across different age groups within the elderly population. Additionally, it explores how specific dietary patterns influence cardiovascular risk, aiming to identify modifiable risk factors that could improve cardiovascular health in this demographic. Specifically, we aim to determine whether certain dietary patterns are associated with a lower risk of CVD among older adults.

## Materials and Methods

### Study design and participants

**Figure 1:**
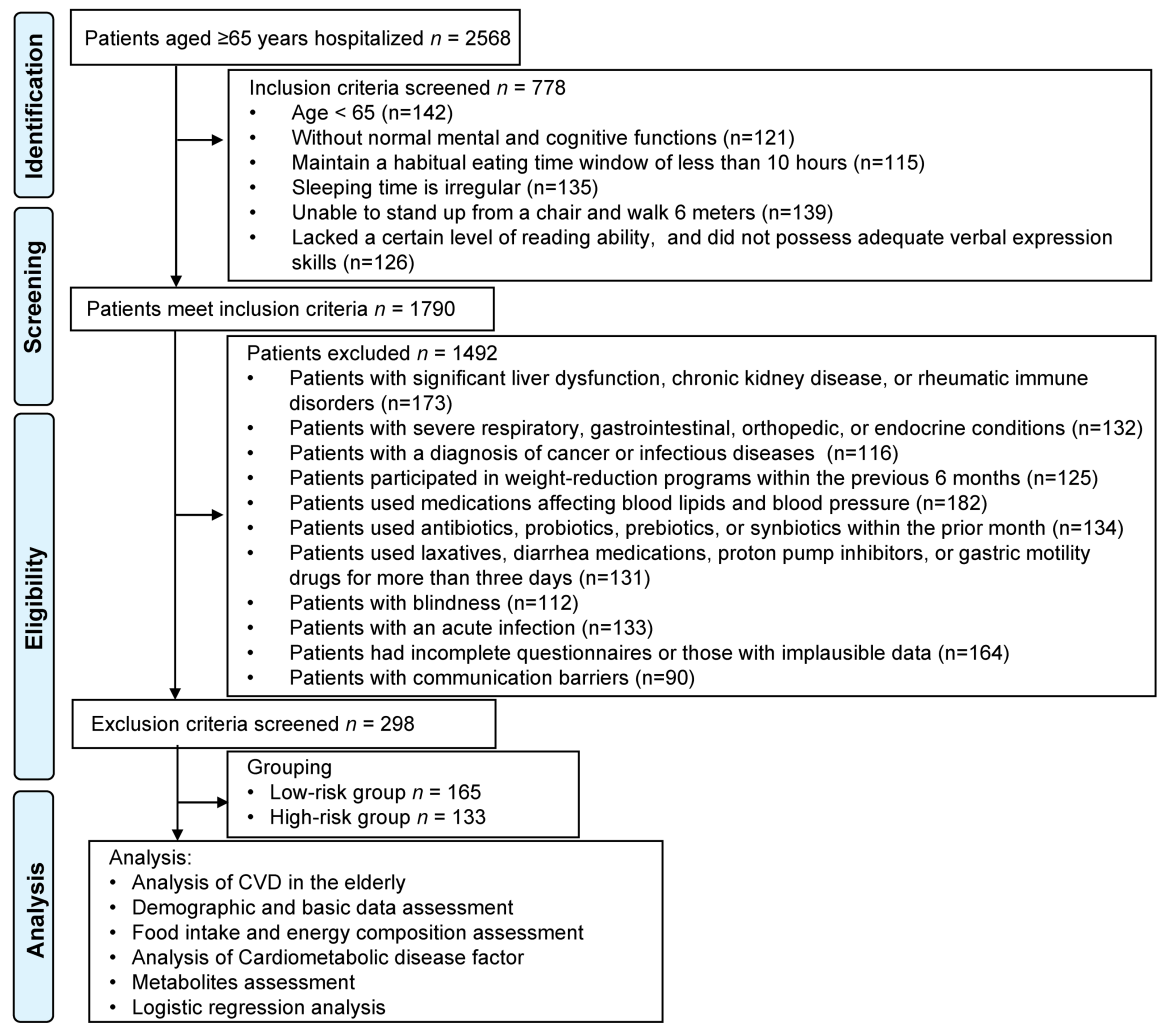
Flowchart of patient selection.

This retrospective study was conducted at Huangshi Central Hospital. The study involved. We analyzed the medical records of 2,568 patients aged ≥65 years hospitalized between 2022 and 2024 to determine the distribution of CVD incidence. From this initial cohort, 298 participants were selected based on stringent inclusion and exclusion criteria for a detailed analysis of dietary patterns and CVD risk. We employed the China-PAR model (Yang et al., 2016) to evaluate the cardiovascular risk among these 298 patients. Subsequently, participants were categorized into low-risk and high-risk groups according to whether their risk exceeded 5.0%. This stratification aimed to investigate the relationship between dietary patterns and CVD in the elderly. Of the participants, 165 were classified into the low-risk group, and 133 were placed in the high-risk group (Fig. 1).

### Inclusion and exclusion criteria

Inclusion Criteria: Participants were eligible for inclusion if they met the following criteria: (1) aged 65 years or older; (2) possessed normal mental and cognitive functions; (3) maintained a habitual eating time window of at least 10 h; (4) adhered to a regular sleeping schedule; (5) demonstrated the ability to stand from a chair and walk 6 m; and (6) had a certain level of reading ability, assessed by independently reading the first page of a questionnaire and accurately answering five randomly selected questions, or possessed adequate verbal expression skills.

Exclusion Criteria: Participants were excluded if they had: (1) significant liver dysfunction, chronic kidney disease, or rheumatic immune disorders; (2) severe respiratory, gastrointestinal, orthopedic, or endocrine conditions; (3) a diagnosis of cancer or infectious diseases; (4) participated in weight-reduction programs within the previous 6 months; (5) used medications affecting blood lipids and blood pressure, such as corticosteroids, hormone replacement therapy, antidepressants, routine use of nonsteroidal anti-inflammatory drugs, or supplements and antacids containing magnesium or calcium; (6) used antibiotics, probiotics, prebiotics, or synbiotics within the prior month; (7) used laxatives, diarrhea medications, proton pump inhibitors, or gastric motility drugs for more than three days; (8) blindness; (9) an acute infection; (10) incomplete questionnaires or those with implausible data, such as reported energy intake below 800 kcal/day or above 5,000 kcal/day; or (11) communication barriers.

### Ethics statement

The Institutional Review Board and Ethics Committee of Huangshi Central Hospital, Affiliated Hospital of Hubei Polytechnic University granted approval for this study. Informed consent was waived for this retrospective study due to the exclusive use of de-identified patient data, which posed no potential harm or impact on patient care. This waiver was approved by the Institutional Review Board and Ethics Committee of our institution in accordance with regulatory and ethical guidelines pertaining to retrospective research studies. The trial adhered to the Declaration of Helsinki and complied with the Good Clinical Practice guidelines as outlined by the International Conference on Harmonisation.

### Experimental approach

Physicians retrieved the medical records of elderly patients hospitalized at our facility between 2022 and 2024. Patients were categorized by age and gender, and the incidence rates of various CVD were calculated for each category. This analysis was used to compare the distribution of CVD among elderly individuals across different ages and genders. From this cohort, 298 patients were selected based on the inclusion criteria. The China-PAR risk assessment model, developed by the National Center for CVD, was employed to estimate the 10-year risk of atherosclerotic CVD (ASCVD) in these patients.

Subsequently, the 298 patients were divided into groups based on their high or low risk of CVD. To examine the relationship between dietary patterns and CVD risk, a questionnaire survey was conducted using the European Prospective Investigation into Cancer and Nutrition (EPIC) questionnaire (Crowe et al., 2011). All participants visited the hospital in groups to complete a three-day dietary questionnaire, recalling their daily diet, including meals and snacks, as required. Upon receiving the questionnaires, physicians provided one-on-one explanations and demonstrations using food models, emphasizing how to estimate food weight and what details to consider. The questionnaire was only administered once participants demonstrated a clear understanding of how to complete it accurately. After the three-day recording period, physicians collected the questionnaires promptly and conducted personal checks to ensure completeness and accuracy.

### Data collection

Two physicians were responsible for entering and exporting data related to outpatient nutrition consultation and guidance management. To ensure the authenticity and accuracy of the information, they cross-verified each other’s entries. Dietary data from the questionnaires were converted into average daily food intake measurements (grams per day). The nutritional analysis was conducted using the China Food Composition Tables (Zhang et al., 2023) to assess nutrient and energy content. Specifically, the food intake data were systematically entered into a standardized electronic spreadsheet (Microsoft Excel 2019; Microsoft Corp., Redmond, WA, USA) which incorporated the nutrient composition codes and conversion factors from the China Food Composition Tables. This allowed for the automated calculation of daily intake for energy, macronutrients, specific fatty acids, cholesterol, added sugars, fiber, and polyphenols based on the reported quantities of consumed foods.

### Blood sample collection and biochemical analysis

Following the completion of the three-day dietary questionnaire, participants underwent an overnight fast, after which four mL of blood was drawn for biochemical analysis. Serum and plasma were separated and analyzed the same day using a Beckman Coulter AU680 Automatic Biochemical Analyzer to assess blood glucose and lipid profiles. Vitamins and metabolites associated with CVD were detected using a high-performance liquid chromatography instrument (EClassical 3200L; Yilite, Dalian, China). Additionally, trace elements were detected using an atomic absorption spectrophotometer (AA800; Jingke Ruida Technology Co., Ltd, Beijing, China).

### ASCVD risk evaluate

Given that all participants in this study were over 65 years old, they were well-suited for short-term cardiovascular risk assessment. Consequently, the China-PAR risk assessment model, developed by the National Center for CVD, was employed to evaluate the 10-year risk of ASCVD (Yang et al., 2016). The factors considered in this assessment included gender, age, blood pressure levels, use of antihypertensive medications, smoking status, family history of CVD, waist circumference, serum total cholesterol levels, and serum high-density lipoprotein (HDL) levels. The evaluation results provide an estimate of an individual’s 10-year risk of developing ASCVD. A 10-year risk of less than 5.0% was classified as low risk, 5.0% to 9.9% as moderate risk, and 10.0% or greater as high risk.

### Statistical analysis

Data analysis was conducted using SPSS 29.0 statistical software (SPSS Inc., Chicago, IL, USA). Categorical data were represented as (n (%)), while continuous data were expressed as x ± s. Categorical variables were compared using the Chi-square test. Continuous variables were compared using *t*-test for normally distributed data or the Mann–Whitney U test for non-normal data, as assessed by the Shapiro–Wilk test. Pearson correlation analysis was used to examine correlations among continuous variables, and Spearman correlation analysis was applied for categorical variables. Univariate and multivariate logistic regression analyses were performed to identify dietary factors independently associated with high CVD risk. Variables with a *p*-value < 0.1 in univariate analysis or those deemed clinically relevant were included in the multivariate model. The multivariable model was adjusted for potential confounding variables. A *p*-value of less than 0.05 was considered statistically significant.

## Results

### CVD in the elderly

The study examined the incidence rates of major CVD in elderly individuals segmented by age groups (65–74, 75–84, and ≥85 years) (Table 1). Ischemic heart disease presented the highest prevalence among the elderly, with its incidence increasing notably with age. Hypertension showed a relatively stable prevalence across all age groups, while arrhythmia’s occurrence increased progressively with advancing age. Conversely, atherosclerosis and vascular disease displayed decreasing trends in incidence as age advanced, indicating varying age-related patterns in CVD prevalence.

Ischemic heart disease exhibited a significantly higher prevalence in males compared to females (Table 2). Similarly, hypertension was more common in males. Arrhythmia showed the most pronounced discrepancy. Atherosclerosis and vascular disease also displayed higher incidence rates in males compared to females. These gender-based differences in disease incidence indicated a potential influence of sex-specific factors on cardiovascular health risks in the elderly population.

### Demographic and basic data

A total of 298 patients including 165 patients with the low-risk group and 133 patients with the high-risk group were included (Table 3). The groups did not significantly differ by gender distribution, age, smoking status, marital status, number of children, monthly income, living situation, education level, history of surgery, hyperuricemia, bronchial asthma, or chronic obstructive pulmonary disease (all *P* > 0.05). Significant differences were found in BMI (*P* = 0.001), waist circumference (*P* = 0.007), current drinking status (*P* = 0.011), diabetes prevalence (*P* = 0.002), and the 10-year ASCVD risk (*P* < 0.001), indicating that the high-risk group presented with higher body mass index (BMI), waist circumference, and alcohol consumption, as well as a greater prevalence of diabetes and markedly elevated ASCVD risk.

**Table 1:** Incidence rates of major diseases in elderly individuals across different age groups.

**Types of diseases**	Incidence rates (%)			
	65∼74	75∼84	≥85	Total prevalence (%)
Ischemic heart disease	34.63	40.08	45.83	37.83
Hypertension	33.17	29.17	31.14	31.18
Arrhythmia	11.06	16.82	18.63	13.17
Atherosclerosis	12.08	9.59	4.15	10.61
Vascular disease	9.29	8.43	5.68	8.72

**Table 2:** Incidence rates of major diseases in elderly individuals across different genders.

**Types of diseases**	Incidence rates (%)	
	Male	Female
Ischemic heart disease	39.01	21.61
Hypertension	33.51	27.69
Arrhythmia	17.67	6.52
Atherosclerosis	12.83	7.29
Vascular disease	12.31	3.29

**Notes.**

The sum of incidence rates exceeds 100% as participants could be diagnosed with more than one cardiovascular disease.

**Table 3:** Baseline characteristics.

**Parameters**	**Low-risk group (*n* = 165)**	**High-risk group (*n* = 133)**	**t/*χ*** ^**2**^	***P***
Male/Female	92 (55.76%)/73 (44.24%)	85 (63.91%)/48 (36.09%)	2.029	0.154
Age (years)	75.21 ± 3.72	74.8 ± 4.11	0.914	0.361
BMI (kg/m2)	23.28 ± 4.65	25.15 ± 5.12	3.303	0.001
Waist (cm)	86.31 ± 13.27	90.57 ± 13.56	2.729	0.007
Current smoking	24 (14.55%)	29 (21.8%)	2.654	0.103
Current drinking	28 (16.97%)	39 (29.32%)	6.448	0.011
Marital status (Married/Others)	151 (91.52%)/14 (8.48%)	119 (89.47%)/14 (10.53%)	0.361	0.548
Number of children			0.216	0.642
≤2	117 (70.91%)	91 (68.42%)		
≥3	48 (29.09%)	42 (31.58%)		
Monthly income (yaun)			0.591	0.442
<5,000	122 (73.94%)	93 (69.92%)		
≥5,000	43 (26.06%)	40 (30.08%)		
Living situation			2.053	0.152
Living alone	20 (12.12%)	24 (18.05%)		
Living with Family	145 (87.88%)	109 (81.95%)		
Education			0.349	0.555
Below high school	105 (63.64%)	89 (66.92%)		
High school and above	60 (36.36%)	44 (33.08%)		
History of surgery	14 (8.48%)	20 (15.04%)	3.128	0.077
Diabetes	4 (2.42%)	15 (11.28%)	9.671	0.002
Hyperuricemia	23 (13.94%)	26 (19.55%)	1.687	0.194
Bronchial asthma	20 (12.12%)	21 (15.79%)	0.835	0.361
Chronic obstructive pulmonary disease	16 (9.7%)	23 (17.29%)	3.736	0.053
the 10-year ASCVD risk (%)	2.3 ± 0.65	10.7 ± 2.43	38.711	<0.001

**Notes.**

BMI Body Mass Index

### Food intake

**Figure 2:**
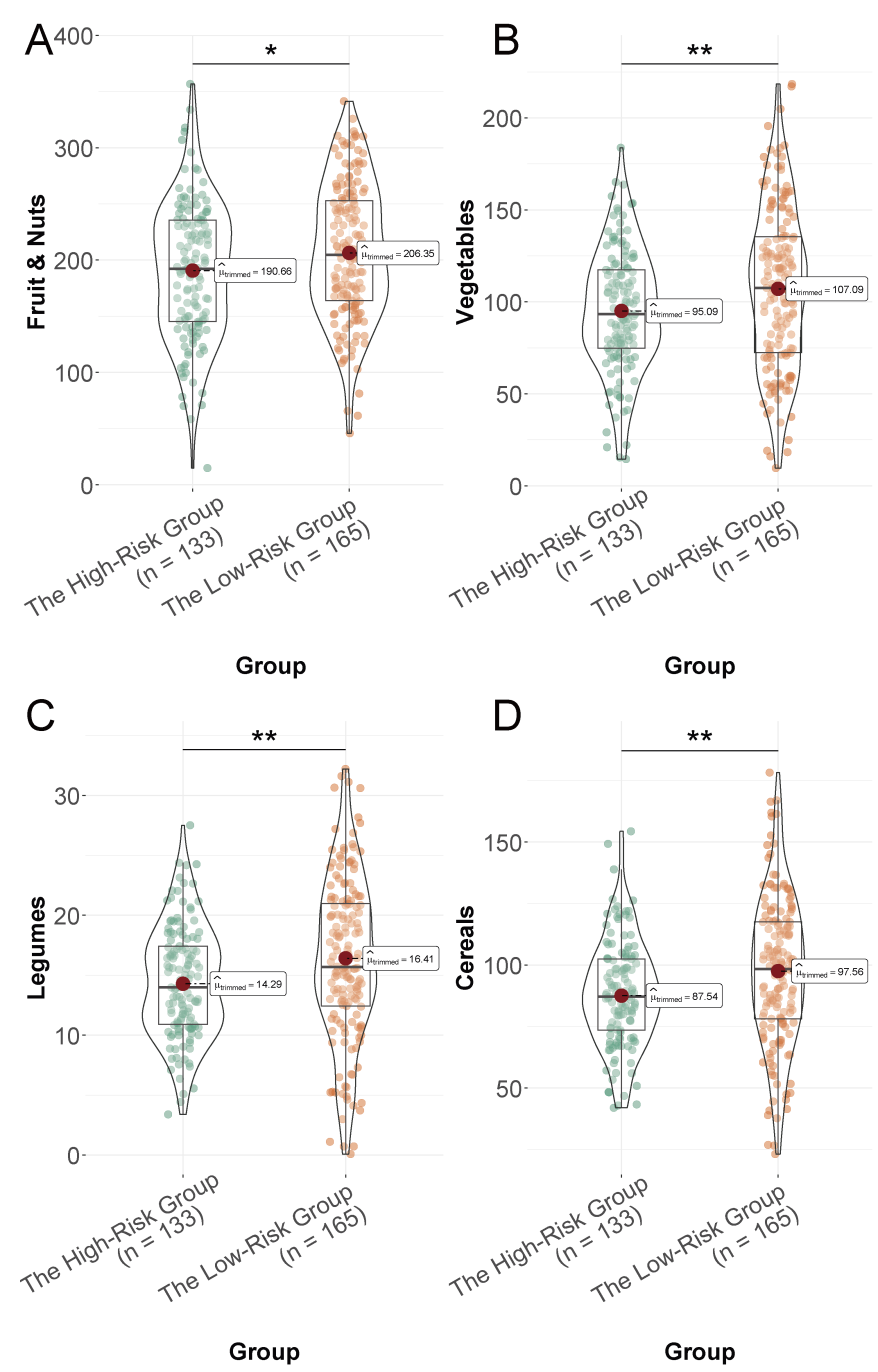
Daily intake of selected food groups (expressed as grams per 1,000 kcal/day) in the low-risk and high-risk groups. (A) Fruit and nuts; (B) vegetables; (C) legumes; (D) cereals. *: *P* < 0.05, ** *P* < 0.01.

**Figure 3:**
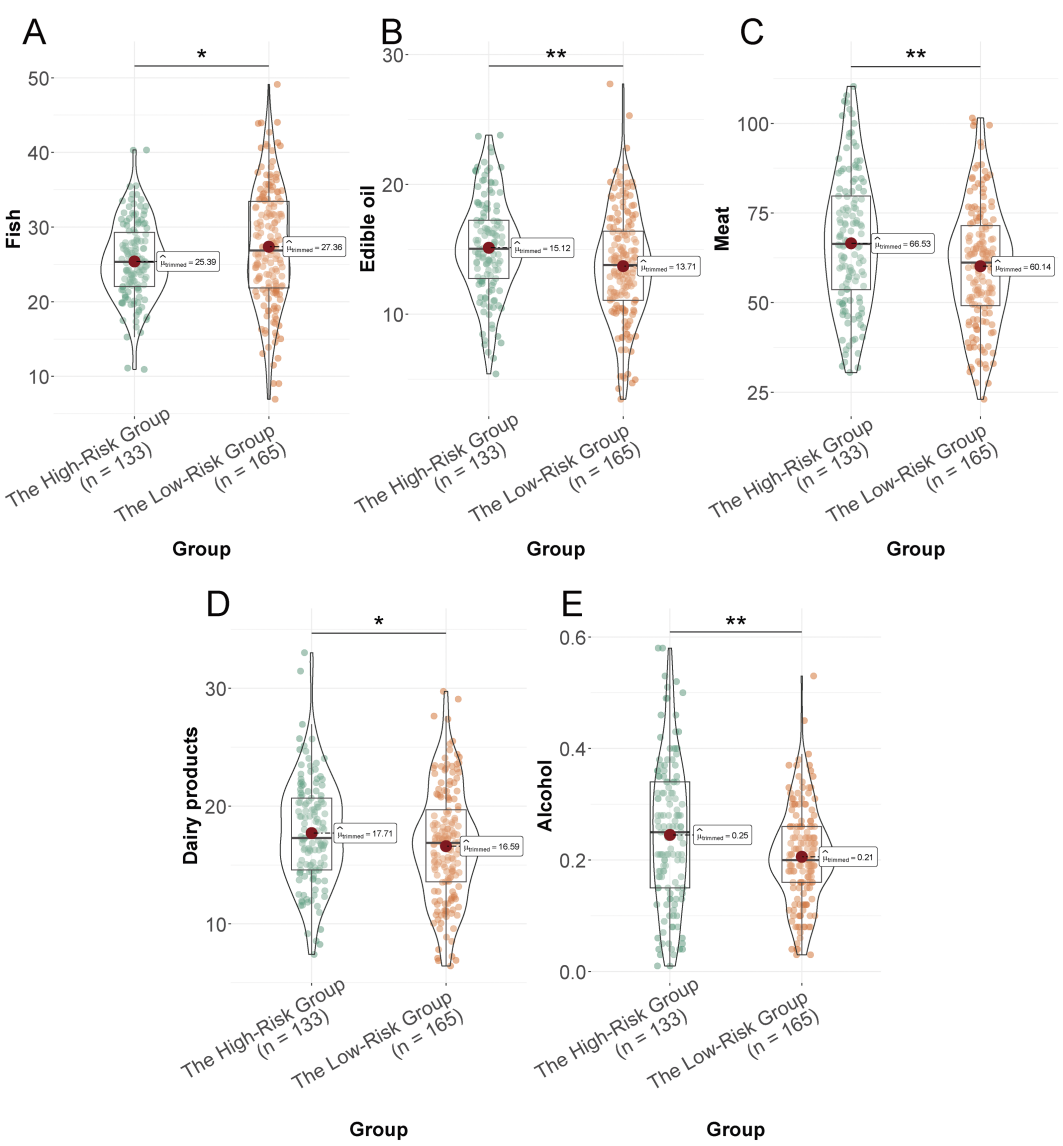
Daily intake of selected food groups (expressed as grams per 1,000 kcal/day) in the low-risk and high-risk groups. (A) Fish; (B) edible oil; (C) meat; (D) dairy products; (E) alcohol. *: *P* < 0.05, ** *P* < 0.01.

The low-risk group had a higher intake of fruits and nuts (*P* = 0.014, Fig. 2A), vegetables (*P* = 0.005, Fig. 2B), legumes (*P* = 0.007, Fig. 2C), cereals (*P* = 0.003, Fig. 2D), and fish (*P* = 0.014, Fig. 3A) compared to the high-risk group. Conversely, the high-risk group had a higher consumption of edible oil (*P* = 0.004, Fig. 3B), meat (*P* = 0.001, Fig. 3C), and alcohol (*P* = 0.002, Fig. 3E). Dairy product consumption showed a marginally significant difference (*P* = 0.05, Fig. 3D), suggesting varied dietary food intake that may impact cardiovascular risk in the elderly.

### Energy composition

The high-risk group had a higher total energy intake (*P* = 0.003), with increased intake of proteins both from animal sources (*P* = 0.031) and overall proteins (*P* = 0.012, Table 4). The low-risk group consumed more vegetable protein (*P* = 0.004) and monounsaturated fatty acids (MUFA, *P* = 0.022), while the high-risk group had higher intakes of lipids (*P* = 0.014), saturated fatty acids (SFA, *P* = 0.009), cholesterol (*P* = 0.003), and added sugars (*P* = 0.012). Carbohydrate intake was slightly lower in the high-risk group (*P* = 0.021). Differences in fiber intake and total polyphenols were not statistically significant (*P* = 0.051 and *P* = 0.342, respectively). Polyunsaturated fatty acid (PUFA) consumption also showed no significant difference (*P* = 0.170). These findings highlight distinct dietary energy composition between the groups, potentially influencing cardiovascular risk profiles in the elderly.

The high-risk group consumed more total energy (*P* = 0.003) and had higher percentages of total protein (*P* = 0.012), specifically from animal sources (*P* = 0.031, Table 5). The low-risk group consumed more vegetable proteins (*P* = 0.004) and monounsaturated fatty acids (MUFA, *P* = 0.022). Conversely, the high-risk group had significantly higher intake of lipids (*P* = 0.014), saturated fatty acids (SFA, *P* = 0.009), cholesterol (*P* = 0.003), and added sugars (*P* = 0.012). The low-risk group had a slightly higher carbohydrate intake (*P* = 0.021). Differences in polyunsaturated fatty acids (PUFA, *P* = 0.170), fiber intake (*P* = 0.051), and total polyphenols (*P* = 0.342) were not significant. These findings underscore different trace elements composition between the groups, which may contribute to cardiovascular risk in the elderly.

**Table 4:** Compare of energy composition of the diet between study groups.

**Variable**	**Low-risk group (*n* = 165)**	**High-risk group (*n* = 133)**	***t***	***P***
Total energy (kcal/day)	1,759.7 ± 452.07	1,923.12 ± 504.5	2.945	0.003
Proteins (% TE)	18.36 ± 3.74	19.37 ± 3.14	2.537	0.012
Proteins Animal sources (% TE)	12.40 ± 2.13	13.10 ± 3.19	2.177	0.031
Proteins Vegetable sources (% TE)	6.67 ± 1.58	6.21 ± 1.21	2.873	0.004
Lipids (% TE)	36.20 ± 6.75	38.45 ± 8.62	2.462	0.014
SFA (% TE)	9.78 ± 2.13	10.63 ± 3.16	2.634	0.009
MUFA (% TE)	19.40 ± 4.82	18.09 ± 4.97	2.306	0.022
PUFA (% TE)	4.60 ± 1.19	4.40 ± 1.26	1.376	0.17
Cholesterol (mg/die)	276.37 ± 73.55	303.17 ± 81.79	2.974	0.003
Carbohydrates (% TE)	46.72 ± 8.73	44.32 ± 9.13	2.313	0.021
Added sugars (% TE)	2.13 ± 0.85	2.41 ± 1.03	2.517	0.012
Fiber (g/1000 kcal/day)	12.28 ± 3.12	11.57 ± 3.06	1.957	0.051
Total Polyphenols (mg)	729.45 ± 236.7	702.17 ± 256.78	0.952	0.342

**Notes.**

SFA Saturated Fatty Acids MUFA Monounsaturated Fatty Acids PUFA Polyunsaturated Fatty Acids % TE Percentage of Total Energy intake

**Table 5:** Compare of trace elements composition of the diet between study groups.

**Variable**	**Low-risk group (*n* = 165)**	**High-risk group (*n* = 133)**	***t***	***P***	**Daily recommendation (adults ≥ 65 years)**
Calcium (mg)	736.3 ± 218.47	817.2 ± 265.27	2.828	0.005	1,200 mg
Sodium (mg)	1,729.2 ± 472.39	1,864.7 ± 513.8	2.367	0.019	<2,300 mg
Potassium (mg)	3,012.7 ± 561.7	3,079.8 ± 611.3	0.985	0.325	4,700 mg
Iron (mg)	8.9 ± 1.31	9.2 ± 1.39	1.881	0.061	8 mg (M), 8 mg (F)
Zinc (mg)	9.37 ± 2.36	10.01 ± 2.34	2.329	0.021	11 mg (M), 8 mg (F)
Selenium (μg)	45.02 ± 6.39	47.26 ± 6.77	2.938	0.004	55 µg
Vitamin A (μg)	769.87 ± 197.3	739.38 ± 174.9	1.394	0.164	900 µg (M), 700 µg (F)
Vitamin E (mg)	15.40 ± 2.39	16.19 ± 2.67	2.682	0.008	15 mg
Vitamin C (mg)	90.79 ± 13.79	85.75 ± 15.67	2.954	0.003	90 mg (M), 75 mg (F)
Vitamin D (IU)	702.47 ± 109.37	733.14 ± 124.8	2.259	0.025	800 IU

**Notes.**

M Male F Female

### Cardiometabolic disease factor

The high-risk group exhibited elevated levels of glycated hemoglobin (A1C, *P* = 0.017) and fasting blood glucose (FBG, *P* = 0.003), indicating poorer glycemic control (Table 6). Total cholesterol (*P* = 0.002) and low-density lipoprotein (LDL, *P* = 0.006) were also higher in the high-risk group. In contrast, HDL levels were lower (*P* = 0.003), and triglycerides (TGs) were elevated (*P* = 0.002) in the high-risk group. Additionally, insulin levels were higher in the high-risk group (*P* = 0.018). These findings indicate that individuals in the high-risk group have a more adverse cardiometabolic profile, which may be associated with increased cardiovascular risk.

**Table 6:** Compare the cardiometabolic disease factor between study groups.

**Variable**	**Low-risk group (*n* = 165)**	**High-risk group (*n* = 133)**	***t***	***P***	**Reference range for adults ≥ 65 years**
A1C (%)	5.15 ± 1.33	5.59 ± 1.78	2.412	0.017	<6.5%
FBG (mmol/L)	4.12 ± 0.65	4.36 ± 0.76	2.974	0.003	4.4–6.1 mmol/L
Total cholesterol (mg/dL)	183.32 ± 29.24	194.35 ± 32.23	3.092	0.002	<200 mg/dL
LDL (mg/dL)	91.28 ± 12.79	95.36 ± 12.41	2.778	0.006	<100 mg/dL
HDL (mg/dL)	62.71 ± 10.1	59.14 ± 10.7	2.955	0.003	>40 mg/dL
TGs (mg/dL)	132.36 ± 15.9	137.69 ± 13.74	3.054	0.002	<150 mg/dL
Insulin (µIU/mL)	22.37 ± 4.27	23.56 ± 4.36	2.382	0.018	2–25 µIU/mL

**Notes.**

A1C Glycated Hemoglobin FBG Fasting Blood Glucose LDL Low-Density Lipoprotein HDL High-Density Lipoprotein TGs Triglycerides

### Metabolites

The high-risk group had elevated levels of trimethylamine N-oxide (TMAO, *P* = 0.042) and secondary bile acids (*P* = 0.002), suggesting alterations in lipid metabolism (Table 7). Additionally, lipopolysaccharides (*P* = 0.003) and uric acid (*P* = 0.003) were higher in the high-risk group, indicating systemic inflammation and altered purine metabolism. The high-risk group also showed increased homocysteine levels (*P* = 0.042), which was linked to cardiovascular dysfunction. Conversely, the low-risk group had higher concentrations of flavonoids (*P* = 0.030) and plant sterols (*P* = 0.016), which were beneficial for cardiovascular health. Choline levels were notably higher in the low-risk group (*P* = 0.001). Short-chain fatty acids (SCFAs, *P* = 0.039) and sulfur compounds (*P* = 0.033) also exhibited significant differences, though more concisely. These findings highlight metabolite variations that may contribute to differing cardiovascular risk profiles in the elderly.

**Table 7:** Compare of metabolites of the diet between study groups.

**Variable**	**Low-risk group (*n* = 165)**	**High-risk group (*n* = 133)**	***t***	***P***
Trimethylamine N-oxide (μM/L)	9.56 ± 3.37	10.36 ± 3.32	2.043	0.042
Short-chain fatty acids (mM)	48.4 ± 6.75	46.82 ± 6.26	2.069	0.039
Secondary bile acids (μM/L)	4.31 ± 1.69	4.95 ± 1.78	3.183	0.002
Lipopolysaccharides (ng/mL)	1.06 ± 0.33	1.18 ± 0.37	2.975	0.003
Uric acid (mg/dL)	7.41 ± 1.38	7.87 ± 1.24	2.967	0.003
Homocysteine (μM/L)	16.15 ± 3.69	17.14 ± 4.54	2.049	0.042
Flavonoids (μM/L)	0.48 ± 0.15	0.44 ± 0.16	2.179	0.03
Choline (μM/L)	13.32 ± 1.27	12.84 ± 1.31	3.217	0.001
Plant sterols (mg/dL)	1.23 ± 0.37	1.13 ± 0.31	2.427	0.016
Sulfur compounds (μM/L)	0.63 ± 0.14	0.59 ± 0.14	2.139	0.033

### Logistic regression analysis

The univariate logistic regression analysis (crude model) identified several dietary patterns significantly associated with a high risk of CVD in the elderly (Table 8). Increased intake of fruits and nuts (OR, 0.995; *P* = 0.015), vegetables (OR, 0.992; *P* = 0.007), legumes (OR, 0.949; *P* = 0.010), cereals (OR, 0.987; *P* = 0.005), and fish (OR, 0.960; *P* = 0.020) were associated with a lower risk of CVD. Conversely, higher consumption of edible oil (OR, 1.090; *P* = 0.004), meat (OR, 1.022; *P* = 0.001), dairy products (OR, 1.052; *P* = 0.050), and particularly alcohol (OR, 24.968; *P* = 0.004) was linked to an increased risk. These findings underscore the impact of specific dietary components on cardiovascular risk in the elderly, highlighting protective effects from plant-based foods and risks associated with animal products and alcohol consumption.

**Table 8:** Univariate logistic regression analysis (crude model) of dietary patterns and high risk of CVD in the elderly.

**Influencing factors**	**Coefficient**	**Std error**	**Wald**	***P***	**OR**	**95% CI**
Fruit & Nuts	−0.005	0.002	2.421	0.015	0.995	0.991–0.999
Vegetables	−0.008	0.003	2.697	0.007	0.992	0.986–0.998
Legumes	−0.053	0.020	2.586	0.010	0.949	0.911–0.987
Cereals	−0.013	0.005	2.836	0.005	0.987	0.978–0.996
Fish	−0.041	0.018	2.320	0.020	0.960	0.927–0.993
Edible oil	0.086	0.030	2.877	0.004	1.090	1.029–1.158
Meat	0.022	0.007	3.199	0.001	1.022	1.009–1.036
Dairy products	0.050	0.026	1.961	0.050	1.052	1.001–1.107
Alcohol	3.218	1.119	2.876	0.004	24.968	2.880–234.199

**Notes.**

Crude model. No adjustments were made for covariates.

Multivariate logistic regression analysis, adjusted for gender, age, BMI, waist circumference, current drinking status, and diabetes prevalence, confirmed that different dietary patterns were independent risk factors for high CVD risk in older adults (Table 9). Intake of fruits and nuts was inversely associated with cardiovascular risk (odds ratio (OR), 0.995; *P* = 0.016), as were vegetables (OR, 0.991; *P* = 0.007), legumes (OR, 0.944; *P* = 0.013), cereals (OR, 0.986; *P* = 0.006), and fish (OR, 0.948; *P* = 0.007), suggesting a protective effect. Conversely, the consumption of edible oil (OR, 1.088; *P* = 0.013), meat (OR, 1.024; *P* = 0.002), and particularly alcohol (OR, 114.950; *P* < 0.001) was positively associated with an increased cardiovascular risk. The association of dairy products with cardiovascular risk was not statistically significant (OR, 1.050; *P* = 0.092). These results emphasize diet’s considerable role in modulating cardiovascular risk, highlighting foods that may confer protection *versus* those that may exacerbate disease risk in the elderly population.

**Table 9:** Multivariable logistic regression analysis (adjusted model) of dietary patterns and high risk of CVD in the elderly.

**Influencing factors**	**Coefficient**	**Std error**	**Wald stat**	***P***	**OR**	**OR CI Lower**	**OR CI Upper**
Fruit & Nuts	−0.005	0.002	−2.413	0.016	0.995	0.990	0.999
Vegetables	−0.009	0.003	−2.721	0.007	0.991	0.985	0.997
Legumes	−0.058	0.023	−2.486	0.013	0.944	0.902	0.988
Cereals	−0.014	0.005	−2.772	0.006	0.986	0.976	0.996
Fish	−0.053	0.020	−2.707	0.007	0.948	0.912	0.985
Edible oil	0.084	0.034	2.476	0.013	1.088	1.018	1.162
Meat	0.024	0.008	3.084	0.002	1.024	1.009	1.039
Dairy products	0.049	0.029	1.683	0.092	1.050	0.992	1.111
Alcohol	4.744	1.305	3.637	<0.001	114.950	8.913	1,482.523

**Notes.**

The model was adjusted for potential confounders including gender, age, BMI, waist circumference, current drinking status, and diabetes prevalence.

## Discussion

In this study, we explored the distribution of CVD incidence rates among the elderly and the relationship between various dietary patterns and CVD risk.

One primary observation from our study was the heightened incidence of ischemic heart disease and arrhythmias, which increase with age. This finding aligns with the established understanding that aging was associated with structural and functional cardiac alterations, including myocardial stiffness, left ventricular hypertrophy, and impaired autonomic regulation (Peters, Sharpe & Proenza, 2020; Wagner et al., 2023; Yan et al., 2021). These age-related changes may predispose individuals to ischemic conditions and arrhythmias, emphasizing the need for targeted interventions to mitigate these risks in older populations. The observed decrease in reported incidence of atherosclerosis and vascular disease in the ≥85 years group likely reflects methodological and epidemiological factors rather than a true biological decline. These may include survival bias (where individuals with severe vascular disease are less likely to survive into very old age), competing risks from other fatal conditions, and potential under-diagnosis or shifting clinical focus in the oldest-old population, rather than a lower actual disease burden.

The gender differences in CVD incidence observed in our study parallel findings from numerous epidemiological studies (Caceres et al., 2022; Suman et al., 2023; Vaccarezza et al., 2020) indicating higher prevalence and severity of CVD in males compared to females. This disparity could be attributed to several factors, including discrepancies in cardiovascular physiology and the protective effects of estrogen in premenopausal women. Furthermore, lifestyle factors such as higher smoking rates, alcohol consumption, and lower health-seeking behaviors observed in males may exacerbate the gender gap in cardiovascular risk (Sun et al., 2023).

Our analysis of dietary patterns revealed compelling associations between specific food groups and cardiovascular risk, highlighting the potential of dietary modifications in managing CVD risk among the elderly. A higher intake of fruits, nuts, vegetables, legumes, cereals, and fish was associated with a reduced cardiovascular risk (Trautwein & McKay, 2020). These foods were rich in essential nutrients, antioxidants, and anti-inflammatory compounds, which may counteract oxidative stress and inflammation, known contributors to atherosclerosis and other cardiovascular conditions (Lim, 2022). The high dietary fiber content in these foods also facilitates lipid metabolism regulation and improves glycemic control, thereby reducing CVD risk factors (Rosenberg, 2022).

Conversely, increased consumption of meat, edible oil, and alcohol was positively correlated with heightened cardiovascular risk. This was consistent with the literature (Feingold, 2000), where diets high in saturated fats, trans fats, and cholesterol were associated with elevated blood lipid levels, weight gain, and increased atherosclerotic plaque formation. In particular, excessive alcohol intake has been linked to systemic inflammation, hypertension, and atrial fibrillation, all of which amplify cardiovascular risk (Roerecke, 2021). Notably, our study also elucidated the critical role of energy balance, as participants in the high-risk group exhibited higher total energy intake and an adverse cardiometabolic profile, marked by increased BMI, waist circumference, and diabetes prevalence. These findings suggest that, beyond nutrient composition, energy imbalance potentially exacerbates CVD risk through obesity-related mechanisms such as insulin resistance, endothelial dysfunction, and altered lipid metabolism.

Furthermore, we observed significant differences in cardiometabolic disease factors and metabolites between the low-risk and high-risk groups, suggesting underlying physiological mechanisms influenced by dietary intake. The elevated glycated hemoglobin (A1C) and fasting blood glucose levels in the high-risk group indicate compromised glycemic control, which, combined with increased LDL and reduced HDL levels, forms a metabolic milieu that fosters CVD development. These biochemical markers were particularly crucial in aging, as the interplay between insulin resistance, dyslipidemia, and inflammation accelerates vascular damage (Bhullar et al., 2024).

A novel aspect of our study is the inclusion of metabolite analysis, which provides potential mechanistic insights. Higher levels of TMAO and secondary bile acids in the high-risk group suggest alterations in gut microbiota metabolism, contributing to different cardiovascular outcomes. TMAO, a metabolite produced by gut bacteria from dietary choline and carnitine, has been implicated in promoting cholesterol deposition in arterial walls and enhancing atherosclerosis (Canyelles et al., 2023). Additionally, secondary bile acids, resulting from bacterial conversion of primary bile acids, can exert cytotoxic effects on endothelial cells (Thomas & Fernandez, 2021). This underscores the emerging role of gut microbiota in cardiovascular health, suggesting that dietary interventions targeting gut microorganisms might offer novel therapeutic avenues (Suriano et al., 2024).

The protective effect of higher concentrations of flavonoids and plant sterols in the low-risk group may be attributed to their antioxidative and lipid-lowering properties. Flavonoids, abundant in fruits and vegetables, have been shown to improve endothelial function and reduce blood pressure *via* nitric oxide production (Yamagata & Yamori, 2020). Plant sterols, structural components of plant cell membranes, can lower plasma cholesterol levels by inhibiting intestinal absorption of dietary cholesterol. These bioactive compounds depict how specific dietary patterns can modify cardiovascular risk factors and outcomes, supporting dietary strategies centered around plant-based foods for cardiovascular health improvement.

Choline, found at higher levels in the low-risk group, was crucial for lipid metabolism, neurotransmitter synthesis, and membrane integrity. Its role in cardiovascular health aligns with its metabolism to TMAO, demonstrating that the impact of dietary components on health outcomes can be multifaceted, with beneficial or adverse effects depending on overall dietary context and individual microbiota composition.

While the association between diet and cardiovascular health is well-established, most large-scale studies focus on general populations or primary prevention. Our study provides a detailed analysis of dietary patterns specifically in a cohort of hospitalized elderly Chinese patients, a group with heightened vulnerability and distinct nutritional needs. By integrating the China-PAR model for precise ASCVD risk stratification with comprehensive dietary and metabolite data, our findings offer a more nuanced understanding of the diet-CVD relationship in this specific demographic. Our study reinforces the importance of personalized dietary recommendations, considering individual metabolic responses, genetic predispositions, and lifestyle factors. Such tailored interventions could effectively manage cardiovascular risk, especially in the elderly, who exhibit diverse health trajectories impacted by accumulated exposures over their lifespan.

However, we acknowledge the limitations of our study. Its cross-sectional design precludes the establishment of causality. The use of a hospitalized patient population may introduce selection bias and limit the generalizability of the findings to the broader community-dwelling elderly. Dietary data, though collected using a standardized questionnaire (EPIC), are subject to recall and reporting biases. Furthermore, the gut microbiota, which influences metabolites like TMAO, was not directly analyzed. Future prospective cohort studies and interventional trials in community-based elderly populations, incorporating longitudinal microbiome analysis, are needed to confirm these associations and elucidate the underlying causal mechanisms.

## Conclusion

In conclusion, our findings indicate significant age-related trends in the prevalence of certain key cardiovascular conditions, with notable gender-based differences. Additionally, specific dietary components were identified as potential risk or protective factors for cardiovascular health in older adults. It highlights the necessity of multifaceted approaches encompassing diet, lifestyle modification, and risk factor management to mitigate cardiovascular risk and promote healthy aging. Future research should focus on longitudinal analyses and interventional studies to establish causality and explore mechanistic pathways, potentially incorporating innovations in microbiome science and nutrigenomics. Emphasizing individual-specific interventions and preventative strategies could substantially enhance cardiovascular outcomes in aging populations, reducing morbidity and associated healthcare burdens.

##  Supplemental Information

10.7717/peerj.20768/supp-1 Supplemental Information 1 data

10.7717/peerj.20768/supp-2 Supplemental Information 2 codebook

10.7717/peerj.20768/supp-3 Supplemental Information 3 STROBE checklist

## Additional Information and Declarations

### Data Availability

The following information was supplied regarding data availability: The raw data and code are available in the Supplemental Files.
