# Supplementary material for: The distribution of incidence rates of cardiovascular diseases in the elderly and the relationship between dietary patterns and cardiovascular risk
Source: PeerJ. 2026 Feb 27;14:e20768. doi: 10.7717/peerj.20768 (PMC12951887; doi:10.7717/peerj.20768)
Supplement: Supplemental Information 3 [file peerj-14-20768-s003.docx]

STROBE Statement—checklist of items that should be included in reports of observational studies

|  | Item No. | Recommendation | Page  No. | Relevant text from manuscript |
| --- | --- | --- | --- | --- |
| **Title and abstract** | 1 | (*a*) Indicate the study’s design with a commonly used term in the title or the abstract | 1 | The distribution of incidence rates of cardiovascular diseases in the elderly and the relationship between dietary patterns and their risk |
|  |  | (*b*) Provide in the abstract an informative and balanced summary of what was done and what was found | 1 | "Background: Cardiovascular diseases (CVD) were leading causes of morbidity and mortality in the elderly. This study investigates the distribution of CVD incidence among older adults and examines the relationship between dietary patterns and associated risks."  "Methods: A retrospective analysis was conducted involving 2,568 patients aged 65 and older hospitalized between 2022 and 2024. Out of these, 298 patients were selected based on specific inclusion criteria for cardiovascular risk assessment using the China-PAR model."  "Results: Ischemic heart disease was most prevalent (37.83%) among the elderly, with increased incidence in males. Higher intakes of fruits, vegetables, legumes, cereals, and fish correlated with reduced CVD risk."  "Conclusion: Higher age and male gender were associated with increased CVD risk, exacerbated by specific dietary patterns. Diets rich in plant-based foods and low in animal products and alcohol effectively reduced CVD risk factors." |
| Introduction | | | |  |
| Background/rationale | 2 | Explain the scientific background and rationale for the investigation being reported | 2 | "Cardiovascular diseases (CVD) remain the leading cause of morbidity and mortality worldwide, and their impact was particularly pronounced in the elderly population."  "Aging was associated with numerous physiological changes that elevate the risk for cardiovascular conditions, including increased arterial stiffness, endothelial dysfunction, and altered autonomic regulation. Despite advancements in medical treatment and preventive measures, the incidence of CVD in older adults continues to rise, underscoring the need for effective strategies to curb this trend."  "One such strategy involves the identification of modifiable risk factors, particularly those related to lifestyle choices, such as diet. Epidemiological studies have repeatedly demonstrated the crucial role of diet in the development and progression of CVD."  "Nutritional status and dietary patterns have been implicated in influencing cardiovascular health through multiple mechanisms, including lipid metabolism regulation, oxidative stress reduction, and inflammation modulation." |
| Objectives | 3 | State specific objectives, including any prespecified hypotheses | 2, 3 | "This study aims to explore the distribution of CVD incidence among the elderly and examine the association between dietary patterns and cardiovascular risk within this population."  "Higher intakes of fruits, vegetables, legumes, cereals, and fish correlated with reduced CVD risk. Conversely, consumptions of meat, edible oil, and alcohol were associated with heightened risk."  "Diets rich in plant-based foods and low in animal products and alcohol effectively reduced CVD risk factors."  "The high-risk group exhibited poorer markers of cardiometabolic health and increased intake of energy, SFA, and cholesterol." |
| Methods | | | |  |
| Study design | 4 | Present key elements of study design early in the paper | 3 | "A retrospective analysis was conducted involving 2,568 patients aged 65 and older hospitalized between 2022 and 2024. Out of these, 298 patients were selected based on specific inclusion criteria for cardiovascular risk assessment using the China-PAR model."  "Participants were stratified into low-risk and high-risk groups for CVD based on a 5% threshold." |
| Setting | 5 | Describe the setting, locations, and relevant dates, including periods of recruitment, exposure, follow-up, and data collection | 4 | "The study involved 2,568 elderly patients, aged 65 years and older, who were hospitalized at our facility between 2022 and 2024."  "Physicians retrieved the medical records of elderly patients hospitalized at our facility between 2022 and 2024."  "All participants visited the hospital in groups to complete a three-day dietary questionnaire, recalling their daily diet, including meals and snacks, as required."  "Following the completion of the three-day dietary questionnaire, participants underwent an overnight fast, after which 4 mL of blood was drawn for biochemical analysis." |
| Participants | 6 | (*a*) *Cohort study*—Give the eligibility criteria, and the sources and methods of selection of participants. Describe methods of follow-up  *Case-control study*—Give the eligibility criteria, and the sources and methods of case ascertainment and control selection. Give the rationale for the choice of cases and controls  *Cross-sectional study*—Give the eligibility criteria, and the sources and methods of selection of participants | 3 | Eligibility Criteria:  "Inclusion Criteria: Participants were eligible for inclusion if they met the following criteria: (1) aged 65 years or older; (2) possessed normal mental and cognitive functions; (3) maintained a habitual eating time window of at least 10 hours; (4) adhered to a regular sleeping schedule; (5) demonstrated the ability to stand from a chair and walk 6 meters; and (6) had a certain level of reading ability, assessed by independently reading the first page of a questionnaire and accurately answering five randomly selected questions, or possessed adequate verbal expression skills."  "Exclusion Criteria: Participants were excluded if they had: (1) significant liver dysfunction, chronic kidney disease, or rheumatic immune disorders; (2) severe respiratory, gastrointestinal, orthopedic, or endocrine conditions; (3) a diagnosis of cancer or infectious diseases; ... (11) communication barriers."  Sources and Methods of Selection:  "We reviewed the medical records of those with complete case histories to analyze the distribution of CVD incidence within this demographic. Based on our inclusion criteria, we selected 298 individuals for further analysis."  Methods of Follow-up:  "Subsequently, participants were categorized into low-risk and high-risk groups according to whether their risk exceeded 5.0%. This stratification aimed to investigate the relationship between dietary patterns and CVD in the elderly."  "To examine the relationship between dietary patterns and CVD risk, a questionnaire survey was conducted using the European Prospective Investigation into Cancer and Nutrition (EPIC) questionnaire." |
|  |  | (*b*) *Cohort study*—For matched studies, give matching criteria and number of exposed and unexposed  *Case-control study*—For matched studies, give matching criteria and the number of controls per case |  |  |
| Variables | 7 | Clearly define all outcomes, exposures, predictors, potential confounders, and effect modifiers. Give diagnostic criteria, if applicable | 4, 5 | "Detailed dietary intake was assessed using the EPIC questionnaire, and blood samples were analyzed for biochemical markers."  "The factors considered in this assessment included gender, age, blood pressure levels, use of antihypertensive medications, smoking status, family history of CVD, waist circumference, serum total cholesterol levels, and serum high-density lipoprotein (HDL) levels." |
| Data sources/ measurement | 8* | For each variable of interest, give sources of data and details of methods of assessment (measurement). Describe comparability of assessment methods if there is more than one group | 5 | "Dietary data from the questionnaires were converted into average daily food intake measurements (grams per day). The nutritional analysis was conducted using the China Food Composition Tables to assess nutrient and energy content."  "Following the completion of the three-day dietary questionnaire, participants underwent an overnight fast, after which 4 mL of blood was drawn for biochemical analysis. Serum and plasma were separated and analyzed the same day using a Beckman Coulter AU680 Automatic Biochemical Analyzer to assess blood glucose and lipid profiles." |
| Bias | 9 | Describe any efforts to address potential sources of bias | 4 | "The Institutional Review Board and Ethics Committee of Huangshi Central Hospital, Affiliated Hospital of Hubei Polytechnic University granted approval for this study. Informed consent was waived for this retrospective study due to the exclusive use of de-identified patient data, which posed no potential harm or impact on patient care." |
| Study size | 10 | Explain how the study size was arrived at | 3 | "Out of these, 298 patients were selected based on specific inclusion criteria for cardiovascular risk assessment using the China-PAR model." |

Continued on next page

| Quantitative variables | 11 | Explain how quantitative variables were handled in the analyses. If applicable, describe which groupings were chosen and why | 5 | "Continuous data were expressed as x ± s. A p-value of less than 0.05 was considered statistically significant." |
| --- | --- | --- | --- | --- |
| Statistical methods | 12 | (*a*) Describe all statistical methods, including those used to control for confounding | 5 | "Data analysis was conducted using SPSS 29.0 statistical software (SPSS Inc., Chicago, IL, USA). Pearson correlation analysis was used to examine correlations among continuous variables, and Spearman correlation analysis was applied for categorical variables. To assess the relationship between dietary patterns and cardiovascular risk, the Log-rank test and Cox regression analysis were employed." |
|  |  | (*b*) Describe any methods used to examine subgroups and interactions | 5 | "Multivariate logistic regression analysis clarified that different dietary patterns were independent risk factors between the high risk of CVD in older adults." |
|  |  | (*c*) Explain how missing data were addressed | 5 | "Participants with incomplete questionnaires or those with implausible data, such as reported energy intake below 800 kcal/day or above 5000 kcal/day, were excluded." |
|  |  | (*d*) *Cohort study*—If applicable, explain how loss to follow-up was addressed  *Case-control study*—If applicable, explain how matching of cases and controls was addressed  *Cross-sectional study*—If applicable, describe analytical methods taking account of sampling strategy | 3 | "This study is a retrospective analysis, so there is no loss to follow-up." |
|  |  | (*e*) Describe any sensitivity analyses |  | Not mentioned in the manuscript. |
| Results | | | | |
| Participants | 13* | (a) Report numbers of individuals at each stage of study—eg numbers potentially eligible, examined for eligibility, confirmed eligible, included in the study, completing follow-up, and analysed | 3 | "The study involved 2,568 elderly patients, aged 65 years and older, who were hospitalized at our facility between 2022 and 2024. Out of these, 298 patients were selected based on specific inclusion criteria." |
|  |  | (b) Give reasons for non-participation at each stage | 3 | "Participants were excluded if they had: (1) significant liver dysfunction, chronic kidney disease, or rheumatic immune disorders; (2) severe respiratory, gastrointestinal, orthopedic, or endocrine conditions; (3) a diagnosis of cancer or infectious diseases; ... (11) communication barriers." |
|  |  | (c) Consider use of a flow diagram |  | Not mentioned in the manuscript. |
| Descriptive data | 14* | (a) Give characteristics of study participants (eg demographic, clinical, social) and information on exposures and potential confounders | 3 | "A total of 298 patients including 165 patients with the low-risk group and 133 patients with the high-risk group were included." |
|  |  | (b) Indicate number of participants with missing data for each variable of interest | 6 | "Participants with incomplete questionnaires or those with implausible data, such as reported energy intake below 800 kcal/day or above 5000 kcal/day, were excluded." |
|  |  | (c) *Cohort study*—Summarise follow-up time (eg, average and total amount) | 7 | "This study is a retrospective analysis, so there is no follow-up time." |
| Outcome data | 15* | *Cohort study*—Report numbers of outcome events or summary measures over time | 8 | "Ischemic heart disease presented the highest prevalence among the elderly, with an overall incidence rate of 37.83%, increasing from 34.63% in the 65–74 age group to 45.83% in those aged ≥85 years." |
|  |  | *Case-control study—*Report numbers in each exposure category, or summary measures of exposure |  |  |
|  |  | *Cross-sectional study—*Report numbers of outcome events or summary measures |  |  |
| Main results | 16 | (*a*) Give unadjusted estimates and, if applicable, confounder-adjusted estimates and their precision (eg, 95% confidence interval). Make clear which confounders were adjusted for and why they were included | 9 | "Increased intake of fruits, vegetables, legumes, cereals, and fish correlated with reduced CVD risk. Conversely, consumptions of meat, edible oil, and alcohol were associated with heightened risk." |
|  |  | (*b*) Report category boundaries when continuous variables were categorized |  | Not mentioned in the manuscript. |
|  |  | (*c*) If relevant, consider translating estimates of relative risk into absolute risk for a meaningful time period |  | Not mentioned in the manuscript. |

Continued on next page

| Other analyses | 17 | Report other analyses done—eg analyses of subgroups and interactions, and sensitivity analyses |  | Not mentioned in the manuscript. |
| --- | --- | --- | --- | --- |
| Discussion | | | | |
| Key results | 18 | Summarise key results with reference to study objectives | 10 | "Higher intakes of fruits, vegetables, legumes, cereals, and fish correlated with reduced CVD risk. Conversely, consumptions of meat, edible oil, and alcohol were associated with heightened risk." |
| Limitations | 19 | Discuss limitations of the study, taking into account sources of potential bias or imprecision. Discuss both direction and magnitude of any potential bias | 11 | "Despite advancements in medical treatment and preventive measures, the incidence of CVD in older adults continues to rise, underscoring the need for effective strategies to curb this trend." |
| Interpretation | 20 | Give a cautious overall interpretation of results considering objectives, limitations, multiplicity of analyses, results from similar studies, and other relevant evidence | 12 | "Diets rich in plant-based foods and low in animal products and alcohol effectively reduced CVD risk factors. These findings underscore the potential of dietary interventions to improve cardiovascular health in the elderly." |
| Generalisability | 21 | Discuss the generalisability (external validity) of the study results | 12 | "These findings underscore the potential of dietary interventions to improve cardiovascular health in the elderly." |
| Other information | |  | | |
| Funding | 22 | Give the source of funding and the role of the funders for the present study and, if applicable, for the original study on which the present article is based |  | Not mentioned in the manuscript. |

*Give information separately for cases and controls in case-control studies and, if applicable, for exposed and unexposed groups in cohort and cross-sectional studies.

**Note:** An Explanation and Elaboration article discusses each checklist item and gives methodological background and published examples of transparent reporting. The STROBE checklist is best used in conjunction with this article (freely available on the Web sites of PLoS Medicine at http://www.plosmedicine.org/, Annals of Internal Medicine at http://www.annals.org/, and Epidemiology at http://www.epidem.com/). Information on the STROBE Initiative is available at www.strobe-statement.org.
